# A Device for Long-Term Perfusion, Imaging, and Electrical Interfacing of Brain Tissue *In vitro*

**DOI:** 10.3389/fnins.2016.00135

**Published:** 2016-03-31

**Authors:** Nathaniel J. Killian, Varadraj N. Vernekar, Steve M. Potter, Jelena Vukasinovic

**Affiliations:** ^1^Laboratory for NeuroEngineering, Wallace H. Coulter Department of Biomedical Engineering at Georgia Tech and Emory University, Georgia Institute of TechnologyAtlanta, GA, USA; ^2^Lena Biosciences Inc., Advanced Technology Development Center (ATDC)Atlanta, GA, USA

**Keywords:** perforated microelectrode array, neurons, brain slice, three-dimensional culture, MEA

## Abstract

Distributed microelectrode array (MEA) recordings from consistent, viable, ≥500 μm thick tissue preparations over time periods from days to weeks may aid in studying a wide range of problems in neurobiology that require *in vivo*-like organotypic morphology. Existing tools for electrically interfacing with organotypic slices do not address necrosis that inevitably occurs within thick slices with limited diffusion of nutrients and gas, and limited removal of waste. We developed an integrated device that enables long-term maintenance of thick, functionally active, brain tissue models using *interstitial* perfusion and distributed recordings from thick sections of explanted tissue on a perforated multi-electrode array. This novel device allows for automated culturing, *in situ* imaging, and extracellular multi-electrode interfacing with brain slices, 3-D cell cultures, and potentially other tissue culture models. The device is economical, easy to assemble, and integrable with standard electrophysiology tools. We found that convective perfusion through the culture thickness provided a functional benefit to the preparations as firing rates were generally higher in perfused cultures compared to their respective unperfused controls. This work is a step toward the development of integrated tools for days-long experiments with more consistent, healthier, thicker, and functionally more active tissue cultures with built-in distributed electrophysiological recording and stimulation functionality. The results may be useful for the study of normal processes, pathological conditions, and drug screening strategies currently hindered by the limitations of acute (a few hours long) brain slice preparations.

## Introduction

To the best of our knowledge, there are no tools which combine long-term (days-long) metabolic support of thick brain tissue preparations via intra-culture perfusion with integrated microelectrode arrays for distributed recording of physiological activity, all in a single system. We designed, fabricated, and validated a device that meets this need. The tool supports interfacing with thick brain tissue sections and provides for long-term electrical stimulation and recording, which will be valuable for studying a wide range of neurobiology phenomena that cannot be understood through acute experimentation lasting only a few hours. Long-term electrical interfacing with thick tissue preparations may supplement many diverse research objectives. For example, spatiotemporal activity patterns that underlie epilepsy have been studied with respect to the structure of the hippocampus *in vitro* (Ferrea et al., 2012) in acute slice experiments. However, performing similar experiments with thicker hippocampal sections and over the period of days may help to gain insight into the anatomical origins of and the development of epileptiform activity. Another application would be to study the structure-function relationships of cortical laminae and local field potentials (Bakker et al., 2009) with greater accuracy by retaining more neurons, neuronal connections, and cellular laminae from the *in vivo* brain, thus overcoming the limitations of thin tissue sections that quickly lose important laminar structures without perfusion. Another important avenue of research might be re-examining knowledge gained from two-dimensional (2-D) dissociated cultures (Jones et al., 2011) and three-dimensional (3-D) cell cultures (Cullen et al., 2011) to further understand the functions of neuron growth in three dimensions. Lastly, brain slices enable studies of the pathophysiology of brain diseases in a tissue context and have been used as neurotoxicological and neuropharmacological screening systems (Noraberg, 2004; Sundstrom et al., 2005; Cho et al., 2007; Humpel, 2015). Long-term maintenance of thick tissue sections would enable repeat dose testing of pharmacological agents modulating neurodegeneration and neurotoxicity, and potentially provide valuable insights into developmental plasticity and repair post-treatment, thus deepening our understanding of regenerative mechanisms and providing avenues for the creation of novel medical treatments.

Present day tools typically resolve only one of the needed requirements for long-term physiological activity recordings from thick preparations, either distributed recordings or perfusion (Huang et al., 2012). Systems which have focused on distributed, multi-electrode array recordings of biological activity from 3-D cell cultures and tissue slices (Stoppini et al., 1997; Gholmieh et al., 2006; Musick et al., 2009; Rajaraman et al., 2011) lacked forced interstitial perfusion for long-term tissue metabolic support and thickness maintenance. This limited the starting thickness of the preparations that could be used for experimentation and/or the duration of studies. On the other hand, systems which focused on perfusion of thick tissue sections (Choi et al., 2007; Rambani et al., 2009; Vukasinovic et al., 2009) were confined to one or a small number of electrodes. While a few electrode sites could serve as a read-out or index of the global state of activity, the reality is typically much more complex; neuronal activity moves in varied and unexpected ways both in the intact brain and in neuronal cultures (Lubenov and Siapas, 2009; Thiagarajan et al., 2010). Indeed, researchers continue to increase the number and density of electrode sites to examine neuronal properties with greater spatial and temporal resolution (Frey et al., 2009; Franke et al., 2012). The utility of multi-channel interfaces is, of course, not limited simply to electrical recording; closed-loop interfaces with stimulation have made use of large numbers of electrodes to produce separable stimulus sets to control neural activity (Bakkum et al., 2008). The study of brain-machine interfaces *in vitro* with clinical applications is emerging and devices such as the one described here may help make this a viable enterprise by increasing electrical interface resolution and enhancing the *in vivo* relevance of the cultured tissue.

For decades, one of the two common methods for acute and organotypic brain slice maintenance has been the interface type chamber where the tissue is seated on a porous membrane at the interface between equilibrated medium and humidified atmosphere (Stoppini et al., 1997; De Simoni and Yu, 2006). Under these conditions, cells in the interior of a tissue slice are typically >200 μm away from basal and apical side of the tissue exposed to medium and air, whereas cells in a mammalian brain are tens of microns away from the capillary blood flow (Karbowski, 2011). For this reason, slices seated on the membrane inevitably decayed, spread laterally and thinned in long-term (days-long) experimentation. However, because this approach is simple, it has been adopted for periodic tissue placement on the MEAs for recordings albeit with thinner (< 400 μm) tissue sections to record shorter term physiological activity (Thiébaud et al., 1997; Berdichevsky et al., 2009). To mitigate the problem of ischemia and necrosis within the slice interior, perforated microelectrode arrays (pMEAs), available from Multi Channel Systems, GmbH (MCS), have been successfully used to provide superfusion and electrical interfacing for thin slice cultures (Boppart et al., 1992; Egert et al., 2005; Stett et al., 2005; Multi Channel Systems MCS GmbH, 2013). In the present work, we adapted pMEAs for surface recording and stimulation to days-long *perfusion* of *thick tissue sections*, with perforations serving as inlet ports for *continuous flow, intra-culture* perfusion.

Using this system, we cultured 0.5–1 mm thick brain slices for up to 5 days *in vitro* (DIV) and 3-D dissociated cell cultures in Matrigel® extracellular matrix (ECM) for up to 6 DIV. Spontaneous and evoked neuronal action potentials were recorded and activity was elicited via electrical or chemical stimulation. Furthermore, cultures were assayed and imaged on the devices to assess survival and thickness. We showed that the system can successfully culture, meet metabolic need, and provide for *in situ* electrical and optical interfacing with thick sections of brain tissue. The novel perfusion system was easy to assemble and straightforward to integrate and interface with the commonly used MEA1060 *in vitro* electrophysiology system from Multi Channel Systems. This work represents a basis for long term distributed electrophysiological recordings from thicker sections of brain tissue, which comprise a greater number of cell layers, and a greater number of live and functionally active cells. We expect that the device may find use in a wide range of applications where there is a need for electrical interfacing with and maintenance of the cellular health of thick sections of brain tissue for many days.

## Methods

### Ethics statement

All experiments were in compliance with the Public Health Service (PHS) Policy on Humane Care and Use of Laboratory Animals, the Eighth Edition of the Guide for the Care and Use of Laboratory Animals, and the Animal Welfare Act. All use of animals and all procedures that used animals were reviewed and approved by the Georgia Institute of Technology Institutional Animal Care and Use Committee (protocol A10042).

### Design and adaptation of perfusion chamber for multi-electrode array interfacing

**Figure 1:**
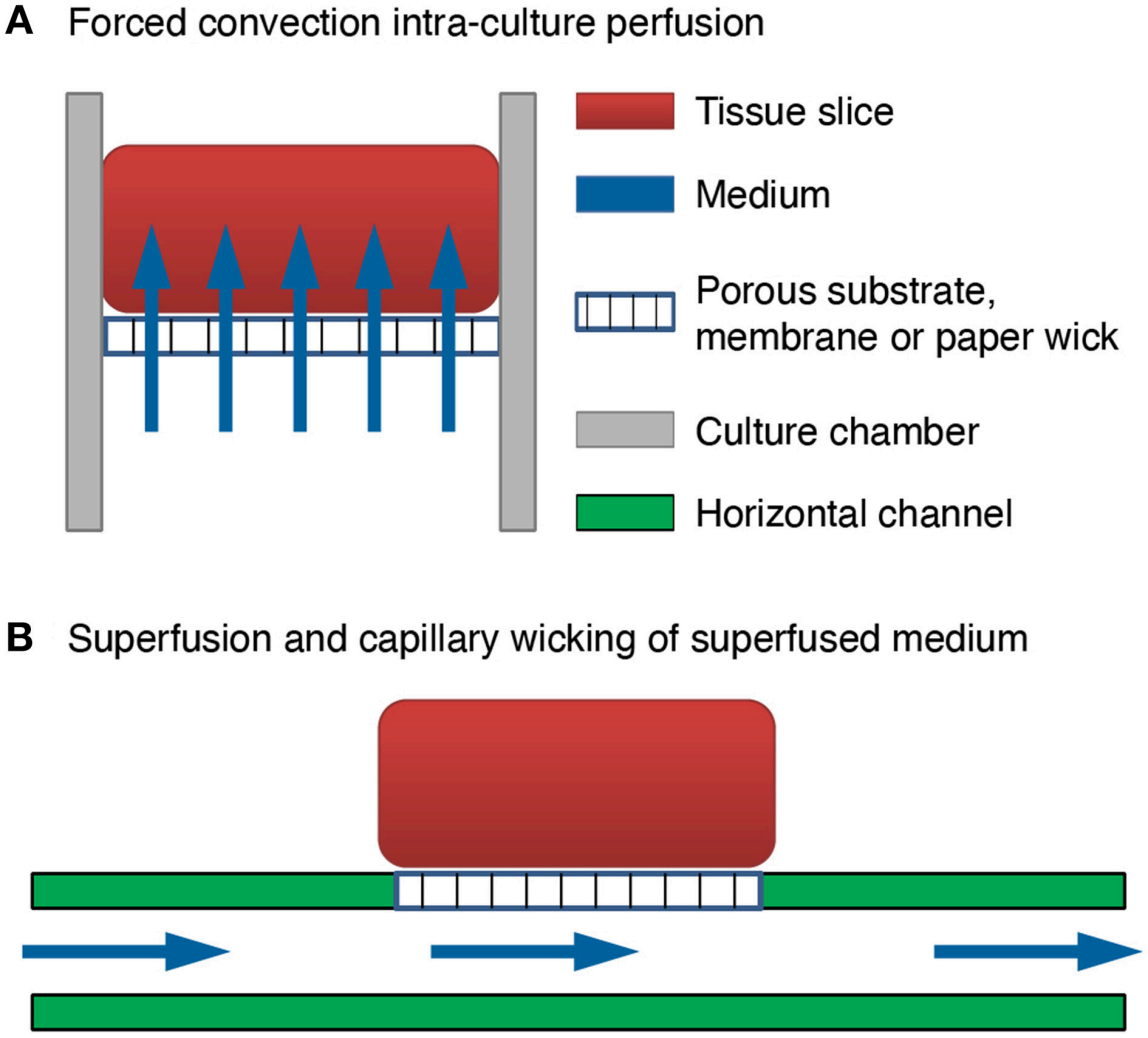
**Comparison of brain slice perfusion methods. (A)** Forced convection intra-culture perfusion. The flow of equilibrated medium is forced to pass through the mass of cells and through the full culture thickness. **(B)** Superfusion and capillary wicking of superfused medium by the tissue slice. The flow of equilibrated medium is not forced to pass through the mass of cells and through the full culture thickness. The resistance to fluid flow through the culture thickness is much higher than the resistance to fluid flow in a channel below the tissue. Flow passes through the channel and the tissue slice wicks the medium from the channel by capillarity.

We invented a method of forced convection interstitial perfusion for long-term maintenance of thick tissue preparations [US Patent 7,855,070 Jelena Vukasinovic and Ari Glezer 2010]. This method is unique and different from previously applied superfusion (extra-tissue perfusion) methods for integrated MEA devices (Thiébaud et al., 1997; Scott et al., 2013). In a forced convection intra-tissue perfusion, equilibrated medium is forced to pass through the mass of cells and throughout the culture thickness (Figure 1A). In superfusion, fluid passes along one or more sides of the tissue, but it is not forced to pass through the tissue due to significantly lower resistance to fluid flow in a channel below the tissue (Figure 1B), or in a pool of medium which surrounds the tissue. Superfusion chambers are typically used for acute slice experiments up to about 12 h (Haas et al., 1979; Zbicz and Weight, 1985). As shown in Figure 1B, in a typical superfusion arrangement, a tissue slice is seated on a porous substrate and wicks medium from the channel below by capillarity. Although medium passes through the channel, there is no flow of medium through the tissue slice. This is because the resistance of tissue slice to fluid flow is inversely proportional to the fourth power of the clearance between the cells (Rambani et al., 2009), which is at the order of tens of nanometers (Thorne and Nicholson, 2006; Syková and Nicholson, 2008), and intra-tissue mass transport remains limited to diffusion. In our invention, flow is forced to pass through the full culture thickness by controlling flow geometry and ensuring adequate culture adhesion. This eliminates paths of low resistance to fluid flow around the culture, and all forced fluid passes through the culture at flow rates that are not deleterious to cells (Rambani et al., 2009).

The device design builds on this previously validated perfusion tool (Rambani et al., 2009; Vukasinovic et al., 2009). The tool enabled long-term maintenance of thick 3-D preparations with enhanced viability by delivering gas-equilibrated medium through the mass of cells with concomitant withdrawal of catabolic waste. We will describe this perfusion tool briefly and then elaborate on its adaptation to accommodate multi-electrode array interfacing in this work.

Using a custom polydimethylsiloxane (PDMS) perfusion chamber with built-in gold mesh for seating the culture, the previous tool provided a slow rate of forced interstitial perfusion through 700 μm thick organotypic brain slice cultures (Rambani et al., 2009). This resulted in higher viability of cells in virtually all cell layers when compared to unperfused slice controls in which the mass transport was limited solely to diffusion of gas and nutrients. Medium was injected into the perfused culture chamber via the gold mesh to which the slice culture was adhered so that there were no paths of lower resistance around the culture. This forced injected medium to pass intra-culture. Medium was withdrawn from circularly cut brain slices via microchannels built into the cylindrical wall of the perfused culture chamber. To enhance gas access to the slice, an aseptic, gas-permeable, and liquid-impermeable membrane was placed around the top face of the chamber and the apical side of the slice culture. Electrical recordings with the previous tool were realized by manually inserting penetrating electrodes into the brain slices; a technique that is serial, difficult, and limited to a few isolated recordings. The present work focuses on an adaptation and improvement of the above described perfusion chamber to enable integrated distributed recording and stimulation functionality.

**Figure 2:**
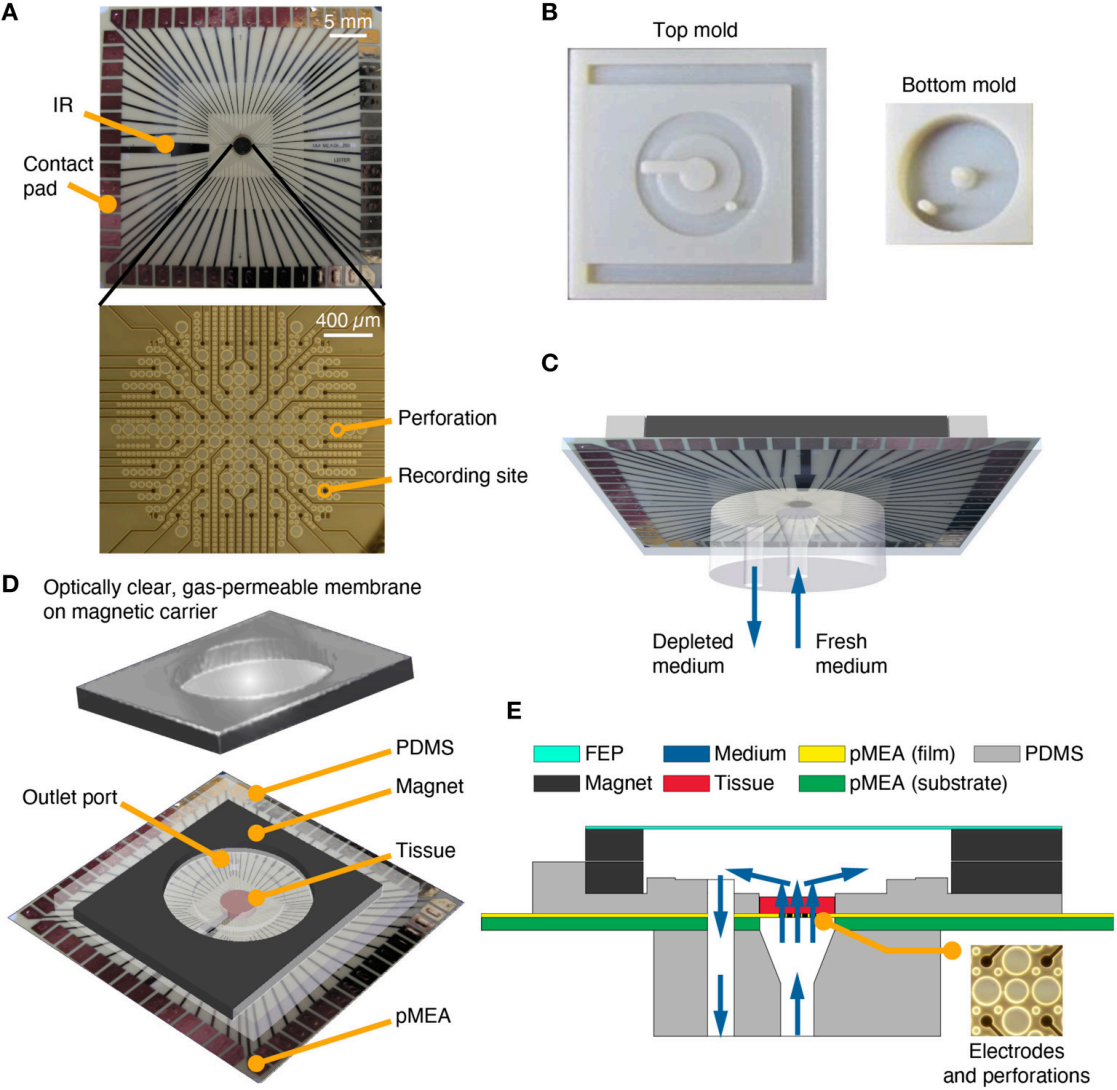
**PDMS-pMEA sandwich design. (A)** pMEA as supplied by the manufacturer, Multi Channel Systems MCS GmbH. The pMEAs have recording sites and perforations, which were used to deliver fresh medium to the tissue culture seated and adhered to the pMEA. The pMEAs have 60 electrodes [59 recording, 1 internal reference (IR)] which were used to electrically record from and stimulate the cultures. **(B)** Plastic molds for the top and bottom pieces of the PDMS-pMEA sandwich. **(C)** The bottom PDMS piece of the PDMS-pMEA sandwich comprising an inlet port to force fresh medium through the tissue and an outlet port to withdraw depleted medium. **(D)** The top PDMS piece of the PDMS-pMEA sandwich comprising perfused culture chamber, a co-axial outer chamber for perfusate withdrawal, and the magnet for attaching the lid. The tissue was seated in the culture chamber directly over the electrode array. **(E)** Vertical cross-section view of the device showing key design components. Medium, depicted by blue arrows, was delivered through the inlet port. It passed through the pMEA perforations and through the tissue in the culture chamber, before it was withdrawn through the outlet port.

Distributed, multi-site recordings are necessary to spatially and temporally resolve and study functional slice activity. Today, such recordings are done using multi-electrode arrays with the added advantages of lower infection risk, improved recording consistency, and unparalleled ease of electrical interfacing with cultures, when compared to manual electrode insertion. This led us to a new design, a perfused recording chamber with an integrated pMEA to enable more consistent, long-term electrophysiological recordings from thicker and healthier tissue models. In a sandwich-like design we integrated pMEAs into unidirectional perfusion chambers (Figure 2). The perfusion methodology was similar to Rambani et al. (2009), except that the pMEA perforations (Figure 2A, bottom) assumed the function of the perfusion openings of the gold mesh. Removable, aseptic, breathable lids were placed on top of the perfused culturing chambers (Figures 2D,E) to seal the devices, maintain humidity, and prevent evaporative losses. Leak tests, evaporation tests, material cytocompatibilty tests, and adhesiveness tests were conducted before finalizing the design to ensure that perfused recording chambers provided a supply of fresh media to the tissues and withdrawal of effluent (depleted medium) in a sterile manner without leakage and evaporative losses.

### Design, fabrication, and fluidic interfacing of PDMS-pMEA sandwich components

Many successful neuronal culturing and interfacing devices have been developed and validated using PDMS (Choi et al., 2007; Rambani et al., 2009; Vukasinovic et al., 2009). PDMS is an excellent choice of material for these applications because of its cytocompatibility, water-tight and water sealing properties, convenience of use, and accuracy in replica-molding of features down to sub-microscopic level, all at a low cost. Here, we replica molded PDMS components, a top and a bottom piece of the perfusion chamber, which sandwiched the pMEA to enable perfusion of thick preparations cultured on the pMEA surface. These PDMS components were made by pouring and curing Dow Corning Sylgard® 184 resin at a 1:10 ratio of catalyst to pre-polymer in custom-made molds fabricated using standard additive manufacturing methods (Figure 2B). A 4.5 mm diameter culture chamber was formed in the top PDMS piece to laterally confine a circular slice of explanted tissue or a 3-D cell culture (Figures 2D,E). A section of the cylindrical wall of the culture chamber opened to a channel that contained medium above the internal reference (IR) ground electrode (Figure 2A, top). The inner, cell culture chamber was built into an outer co-axial chamber that was seated over the withdrawal port made in the pMEA (Figures 2D,E). The flow through the culture residing in the inner culture chamber was unidirectional, bottom-to-top (Figures 2C,E) and the wall of the outer co-axial chamber was raised slightly above the culture chamber to allow uniform withdrawal of perfusate from the culture chamber (this feature can be seen in Figure 2E). The top and bottom components of the PDMS sandwich were sealed to the pMEA using a 200 μm thick double-sided silicone tape (Scapa 702) that enables water-tight sealing and a high-strength bonding of silicone substrates to other silicone substrates and other difficult to bond surfaces. The precise tape layouts conformed to that of the bottom side of the top PDMS component, and the top side of the bottom PDMS component, respectively. These layouts were cut using a computer-controlled automated cutter (Black Cat® Cougar). The bottom piece of the PDMS sandwich was replica molded to contain two vertical circular channels, a center channel and a side channel (Figure 2C). The center channel was made to interface with the perforations on the pMEA and served as an inlet port. The side channel was made to interface with the outer co-axial chamber, surrounding the culture chamber; thus creating an outlet port for withdrawal of perfusate (Figures 2C,D). The top side of the vertical center channel was made wider, with a diameter equal to that of the inner culture chamber, to allow laminar flow of equilibrated medium to uniformly permeate all perforations on the pMEA. The bottom side of both the vertical center channel and the vertical side channel enabled facile insertion and leak-proof interfacing with barbed tube fittings. To prevent leaks and increase wet strength, a thin layer of PDMS was added and cured around the barbed connectors and the edges of PDMS–pMEA interfaces.

Perforations surrounded recording sites over a circular recording region measuring ~2 mm in diameter (Figure 2A, bottom). These perforations served as flow entry ports into the culture. The pMEA porosity in the electrode region was ~63%. To form an outlet port in the pMEA, a hole measuring 1 mm in diameter was drilled through the polyimide pMEA array film and through the ~1-mm thick pMEA carrier substrate (glass or ceramic) near site “81” (the upper right corner of the 8 × 8 MEA grid; Figure 2A). The outlet port location was chosen to minimize damage to the electrode array. Leads that were unavoidably damaged by drilling, typically 3–5 channels in the upper right corner of the array, were connected to ground through the preamplifier (a custom MEA layout could be designed to accommodate this port without sacrificing channels).

**Figure 3:**
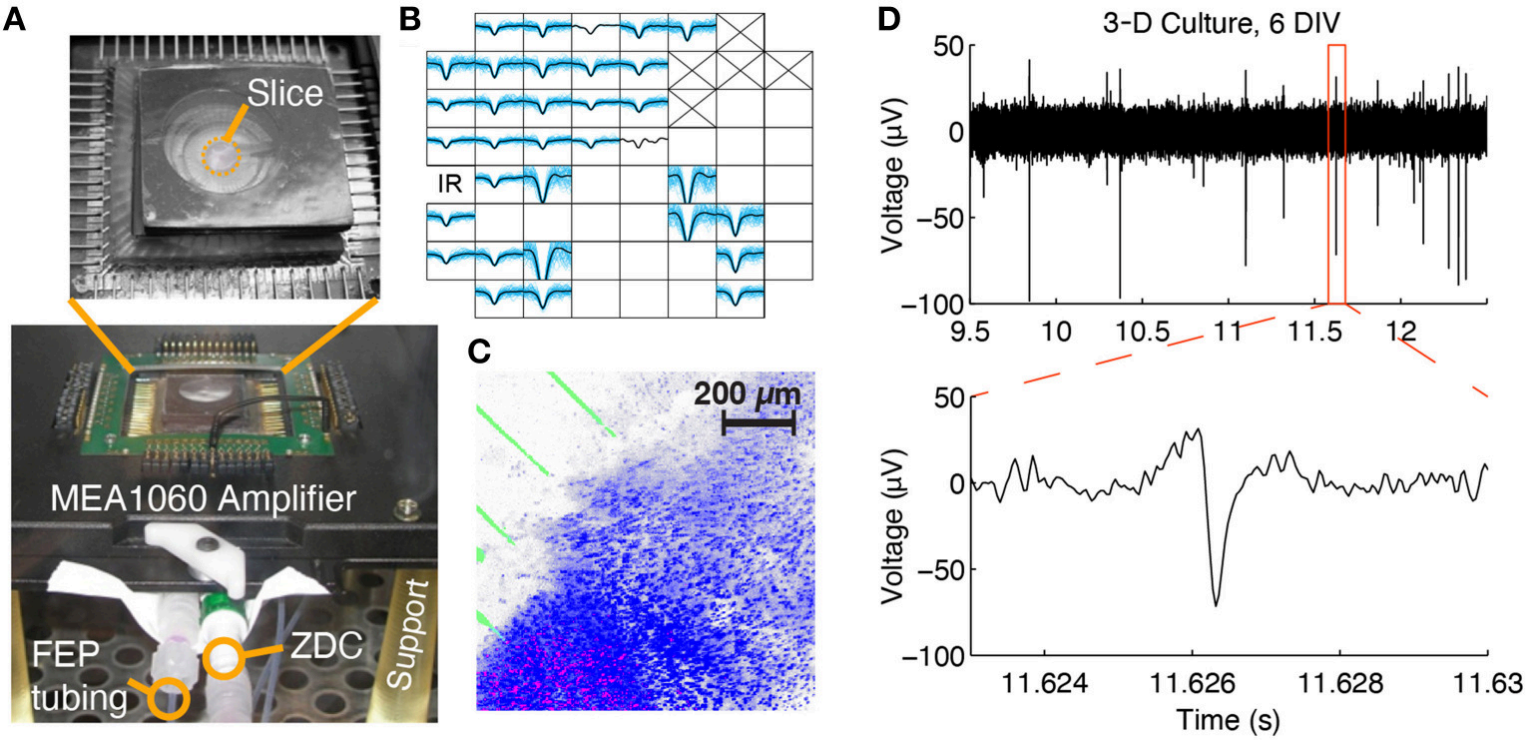
**Multi-channel electrophysiological recording and confocal imaging of perfused slice cultures. (A)** A perfused slice is shown on a pMEA seated in 60-channel preamplifier. The FEP tubing, zero-displacement connector (ZDC), and support legs can be seen. **(B)** Example spontaneous spike waveforms recorded from a slice at 1 DIV during a 2 min recording (blue: individual spikes, black: average waveform). Each plot box represents one channel and the boxes are arranged according to the array geometry. Each plot has a horizontal limit of −400 to 600 μs from the spike trough and a vertical limit of −100 to 50 μV (some waveforms have been clipped so that smaller waveforms are still visible). Channels marked with an “X” were sacrificed to create the medium withdrawal port and blank channels had no spikes. **(C)** Confocal 3-D rendering of a perfused brain slice seated in the perfusion chamber at 1 DIV. Blue: Hoechst 33,342 dye, stained all cell nuclei; Red: Propidium Iodide, stained dead cell nuclei; Green: electrode traces. **(D)** Raw voltage trace containing spikes recorded from a 3-D culture at 6 DIV.

The PDMS-pMEA sandwich was connected to a syringe pump (KD Scientific 260) that carried opposing, infusion and withdrawal syringes on a single drive. The pump enabled infusion of nutrients into the culture chamber and withdrawal of perfusate at an equal flow rate of 28 μL per hour. The device-to-pump interfacing was realized by a combination of tubing. First, 8-cm long sections of flexible tubing (Tygon-2475 tubing with thick walls to limit evaporative loss of medium during perfusion) were attached to barbed connectors and inserted into the PDMS ports. Flexible tubing enabled easy insertion and removal of barbed connectors and virtually stress-free device positioning and interfacing with the preamplifier. Next, flexible tubes were connected to stiff fluorinated ethylene propylene (FEP) tubes (0.5 mm ID, 1.6 mm OD, Upchurch Scientific) using zero-fluid-displacement connectors (ZDCs; RyMed Technologies, Inc.; Figure 3A). ZDCs prevented air entry into the system and pressure surges on the cultures during system setup and priming. Moreover, by using short lengths of flexible tubing attached to ZDCs, the devices could be temporarily disconnected from or connected to the syringe pump for transport. Lastly, stiff tubes were interfaced with 30 mL polypropylene infusion and withdrawal syringes using LuerTight fittings (Upchurch Scientific). In unperfused control cultures, Tygon tubes were filled with medium and then plugged with luer-lock end-caps.

The PDMS components were designed to be thin. The top piece of the PDMS sandwich measured less than a few millimeters in thickness to facilitate *in situ* culture imaging (on the pMEA) and to enable effective interfacing with the MEA1060 preamplifier from Multi Channel Systems. The bottom piece of the sandwich was designed to easily fit in the preamplifier stage with sufficient tolerance for straightforward alignment, secure positioning, and good electrical contact between contact pads and preamplifier pins. To further ease positioning of the device, the central hole of the MEA1060 base was enlarged with a CNC mill to 25.4 mm in diameter. Custom 3-D printed legs (FullCure720 material) provided clearance for the tubing to pass underneath.

### Novel magnetic components for the attachment of breathable lids to perfused pMEA recording chambers

We invented a method to use hydrophobic membranes to seal culture chambers in a way that permits gas exchange, prevents evaporative losses of medium, and reduces the risk of infection [US Patent 6,521,451 Potter 2001]. Prior to the present work, this was implemented by attachment of a gas-permeable FEP membrane to a plastic ring, which was in turn attached to a glass ring serving as the culture well on Multi Channel Systems MEAs and other MEAs of a similar design (Potter and DeMarse, 2001). To adapt this method to the thin PDMS-pMEA sandwich, we attached FEP film (DuPont) to a 2.5 mm thick sheet magnet (ferrite-bonded with synthetic rubber) that was cut to mate with another 1.5 mm thick sheet magnet (Figures 2D,E). The latter was built-into the top piece of the PDMS sandwich (Figure 2D). We found that a 100 μm thick FEP membrane prevented evaporative losses of medium, whereas 50 μm or thinner FEP film led to small but detectable evaporative losses of medium. The key advantage of magnetic or magnetic/magnet receptive lids and components in this design is that they enabled one to “slide” the gas permeable FEP membrane over the PDMS-pMEA sandwich when the cultures were seated into the chambers. This prevented air entrapment into the culture chamber and the resultant pressure surge on the culture caused by a lid pushed top-down. Instead, sliding the lid horizontally over the chamber allowed air to evacuate the chamber without pressurizing the cultures. Hence, magnetic lid interfacing enabled surge-free culture setup, priming and access to cultures as needed (e.g., for media exchanges in unperfused control cultures or addition and aspiration of test compounds). Lastly, since magnetic interfaces provided a tight and leak resistant seal even when the FEP membrane was between them, the lids could be flipped so that the FEP membrane was further (Figure 2E) or closer to the top of the culture which is important for *in situ* culture imaging (through the transparent FEP membrane).

### Tissue culturing and plating

The experiments were performed using both brain slices and dissociated 3-D brain cell cultures in a Matrigel® extracellular matrix (ECM). Brain slices were cultured for up to 5 days and 3-D cell cultures were cultured for up to 6 days. We performed four experimental runs with slices and two experimental runs with 3-D cultures. All slices and 3-D cultures were prepared using the same respective protocols outlined in this section. Not all cultures were used in all experiments, for example, some cultures were used only for imaging to assess viability. Please refer to the sample sizes given in the text and figure legends for each of the analyses performed.

Prior to plating of either culture type, the devices were sterilized by 70% ethanol and ultraviolet-C exposure. External tubes and connectors were steam autoclaved. The pMEA surfaces were treated by glow discharge (EMS-100 glow discharge unit set at 25 mA for 60 s), followed by application of poly-D-lysine (0.1 mg/mL) and then laminin (1 mg/mL) coatings to enable long-term tissue adhesion. The tissue was held in place entirely by way of the coatings on the pMEA and the PDMS walls of the culture chamber. For each coating step, the short flexible tubing was primed with medium and the coating solutions were left in the culture chambers for at least 1 h. After an experimental run, the devices were cleaned and later re-used. Cleaning was performed by gently rinsing with distilled water and soaking in a digestive enzyme solution to remove adhered proteins. When not in use, the devices were allowed to dry and sit in a covered container. Each device underwent at least four experimental runs.

Coronal sections of the brain (4.5 mm diameter, and either 500 or 1000 μm thick) were obtained from Sprague-Dawley rat pups (Charles River; ages P1-9) after euthanasia by isoflurane anesthesia and rapid decapitation. Slices were cut to include regions of the neocortex, hippocampus, and the thalamus. For consistency, sections were taken, from pups, from a region that corresponds to approximately 3 mm posterior to bregma in the adult rat (Paxinos and Watson, 1998). Brains were sliced by a chopping motion with a razor blade either using a brain matrix for 500 μm thick slices or a McIlwain tissue chopper for 1000 μm thick slices. Slices were made in under 1 min whether they were manually chopped or machine-chopped. A 4.5 mm diameter tissue disc was punched out using a disposable biopsy tool (Acuderm, Inc.). Circular slices were then placed in the culture chambers of the pMEAs using a wide-opening transfer pipette (Figure 3A). Both perfused and unperfused slices were plated onto pMEAs and cultured for up to 5 days *in vitro*. Medium composition was 41.4% MEM (no L-glutamine) + 31.4% deionized H_2_O + 2.1% HBSS + 20.7% horse serum + 0.8% D-glucose + 0.8% antibiotic/antimycotic + 0.4% GlutaMAX + 2.4% buffering agents (0.2% NaHCO_3_ + 4.96 mM tris base + 5.6 mM HEPES) and sterile filtered; 294 mOsm/kg (Stoppini et al., 1997; Gähwiler et al., 2001).

3-D brain cell cultures were 2:1 neuron-glia cell ratio co-cultures in 8 mg/mL protein Matrigel® (Cullen et al., 2007) and were plated in 500 or 1000 μm thickness. Perfused 3-D cell cultures were plated onto pMEAs. Some unperfused 3-D cell culture controls were maintained in identical culture chambers (as were those in the pMEA devices) mounted on glass slides. For 3-D cell cultures medium composition was Neurobasal® + 2% B-27 + 1% G-5 + 0.5 mM L-glutamine + 1% antibiotic/antimycotic. The 3-D cell cultures were maintained for 6 days in each of the two experimental runs. Cells were plated at a density of 2500 cells per μL, with about 40,000 cells in a 1000 μm thick culture and 20,000 cells in a 500 μm thick culture.

For each perfused culture, medium was continuously infused into the culture chamber and an equal volume of effluent withdrawn from the device (Figure 2C). Typically two cultures, each seated in a preamplifier, were perfused simultaneously using a single syringe pump. One slice culture in a preamplifier is shown in Figure 3A. For all experiments the flow rate was 28 μL/h. This rate corresponded to ~3.5 culture volume exchanges per hour for 500 μm thick cultures and ~1.8 culture volume exchanges per hour for 1000 μm thick cultures. The medium was equilibrated with the incubator environment (35°C, 21% O_2_, 5% CO_2_,65% RH) before loading into infusion syringes. The entire system was primed with equilibrated medium and visually inspected to ensure absence of air bubbles. Immediately before placing the slices or 3-D cultures, the medium in the chamber was withdrawn with a micropipette. After the slice or 3-D culture was placed, the tissue was allowed to sit in the incubator for up to 30 min to promote adhesion. Next, 200 μL of medium was manually added on top of the tissue with a micropipette and perfusion was initiated. The culture lid was kept in place at all times, except when accessing the chamber, for example, to add the initial medium. The pump was placed outside of the incubator and there was sufficient length of tubing inside the incubator to warm the media before reaching the culture. Unperfused control cultures underwent the same preparation procedures and maintenance as their respective perfused cultures. They were kept in the same incubator, and used the same medium. The medium above unperfused cultures (200 μL) was exchanged manually by a micropipette every 12 h, for the duration of the experiments.

### Recording and stimulation

Recordings were made via pMEA substrate microelectrodes at the bottom surface of the cultured tissue using a modified MCS 60-channel preamplifier designed to interface with upright microscopes (Figure 3). Spontaneous and evoked electrical activity were recorded using MEABench software (Wagenaar et al., 2005). Stimuli, designed to effectively elicit activity (Wagenaar et al., 2004), were delivered using the RACS system (Wagenaar and Potter, 2004). The SALPA stimulus artifact recovery program was used to record as early as few milliseconds after stimulus delivery (Wagenaar and Potter, 2002). Raw signals were filtered offline with a 200–8000 Hz 4th-order Butterworth band-pass filter to detect spikes for firing rate analyses Filtering was performed using zero-phase shift digital infinite impulse response filtering in MATLAB. For each recording in the absence of electrical stimulation, spikes were detected using a constant voltage threshold of 4.25 times the standard deviation of the raw signal sampled at 25 kHz and digitally filtered. Firing rates for slices and 3-D cultures, both perfused and unperfused, were examined over the culturing periods using this spike detection method. To examine responses locked to electrical stimulation, SALPA-filtered data was used and any negative-going spikes with a trough below −3 standard deviations were considered. Spikes occurring within 1 ms after a detected spike were discarded. Noise was further reduced by removing artifactual waveforms. By visually examining time traces of the recordings near the times of stimulus application, we observed large and monophasic waveforms that appeared to be associated with stimulation: these were chiefly voltage excursions that saturated the amplifiers. Therefore, exclusion procedures were developed and then consistently applied to all recordings to remove these erroneous waveforms from the data. We excluded waveforms that were strictly monophasic, waveforms with unusually sharp peaks (a slope at the peak of more than 7 × 10^5^ μV/s), and waveforms that occurred with a time delay of < 1 ms on more than 15% of the channels with recorded spikes. Time-frequency analyses of stimulus-evoked signals were performed using the multitaper method and non-parametric statistics (Thomson, 1982; Maris and Oostenveld, 2007; Oostenveld et al., 2011).

Medium containing 20 mM KCl was used to depolarize cultures and chemically stimulate activity. Studies were done with the cultures placed in the preamplifier by manually exchanging the medium above the cultures using micropipette. The magnetic lids made this procedure easy to perform.

### Staining, imaging, image processing, and outcome measures

On the final day of each study, cultures were stained with Hoechst 33,342, Calcein AM (or Fluo-5f AM), and Propidium Iodide and imaged using an upright confocal laser-scanning microscope (CLSM; Zeiss LSM 510) with an EC Plan Neofluor 10x/0.3 N.A. objective at 364 nm, 488 nm, and 543 nm excitation for the respective dyes. Devices were placed directly on the microscope stage and z-stack images through the culture thicknesses taken *in situ*. Images were processed and quantified using custom MATLAB cell counting code and Zeiss ZEN software. The culture thickness was estimated by taking the vertical scan from the pMEA substrate (imaged with transmitted light) to the last slice in the z-stack where stained cells were visible. Because of imaging and staining limitations associated with the use of thick cultures, we could not stain and image through their entire thickness. First, to maintain procedural consistency and adequately compare perfused and unperfused cultures, the dye solution was added manually to the top of the cultures relying on passive diffusion for staining. Next, the top-down depth of imaging of fluorescently labeled cells was limited to about 400 μm thickness in 3-D cell cultures and 200 μm thickness in brain slices. However, we measured electrophysiological activity of the cells on the pMEA which corroborated the presence of live and functionally active cells at the bottom side of cultures and confirmed good contact between cells and electrodes required for these measurements (Henze et al., 2000; Claverol-Tinture and Pine, 2002). Because of the imaging depth limitations associated with confocal microscopy, we were not able to both record from and image the same cells. Future studies may benefit, however, from techniques developed to increase the fluorescence imaging depth in brain tissue (Theer et al., 2003; Levene et al., 2004).

### Brain slice culture staining, imaging, and image processing

In slice cultures, the nuclei of all cells (live and dead) were labeled with Hoechst 33,342. Propidium iodide (PI) counter-stain was used to label dead cell nuclei. To quantify the number of live vs. dead cells, cultures were imaged through a sample of the volume by two-channel confocal microscopy; “red” channel for PI fluorescence, and a “blue” channel for Hoechst fluorescence. As an example, a three-dimensional reconstruction of a stained and imaged slice is shown in Figure 3C. The live vs. dead imaging process provided two separate culture images at each z-location: an image showing dead cell nuclei (stained by PI), and an image showing nuclei of live and dead cells (stained by Hoechst). All images in a stack were thresholded by selecting pixels with intensities above the 70th percentile for each image and for each channel (red and blue). To aid in discriminating stained nuclei, the watershed transform was applied to distance transformed binary images. Cells were then identified as 4-connected pixel neighborhoods. Only cells with areas between 5 and 500 ${\text{μm}}^{\text{2}}$ were considered to reduce erroneous cell identification due to noise or accumulations of dye. We then estimated cell survival as the density of live cells in the imaged volume: the number of live cells (cells with blue but not red staining) per μL.

### 3-D brain cell culture staining, imaging, and image processing

**Figure 4:**
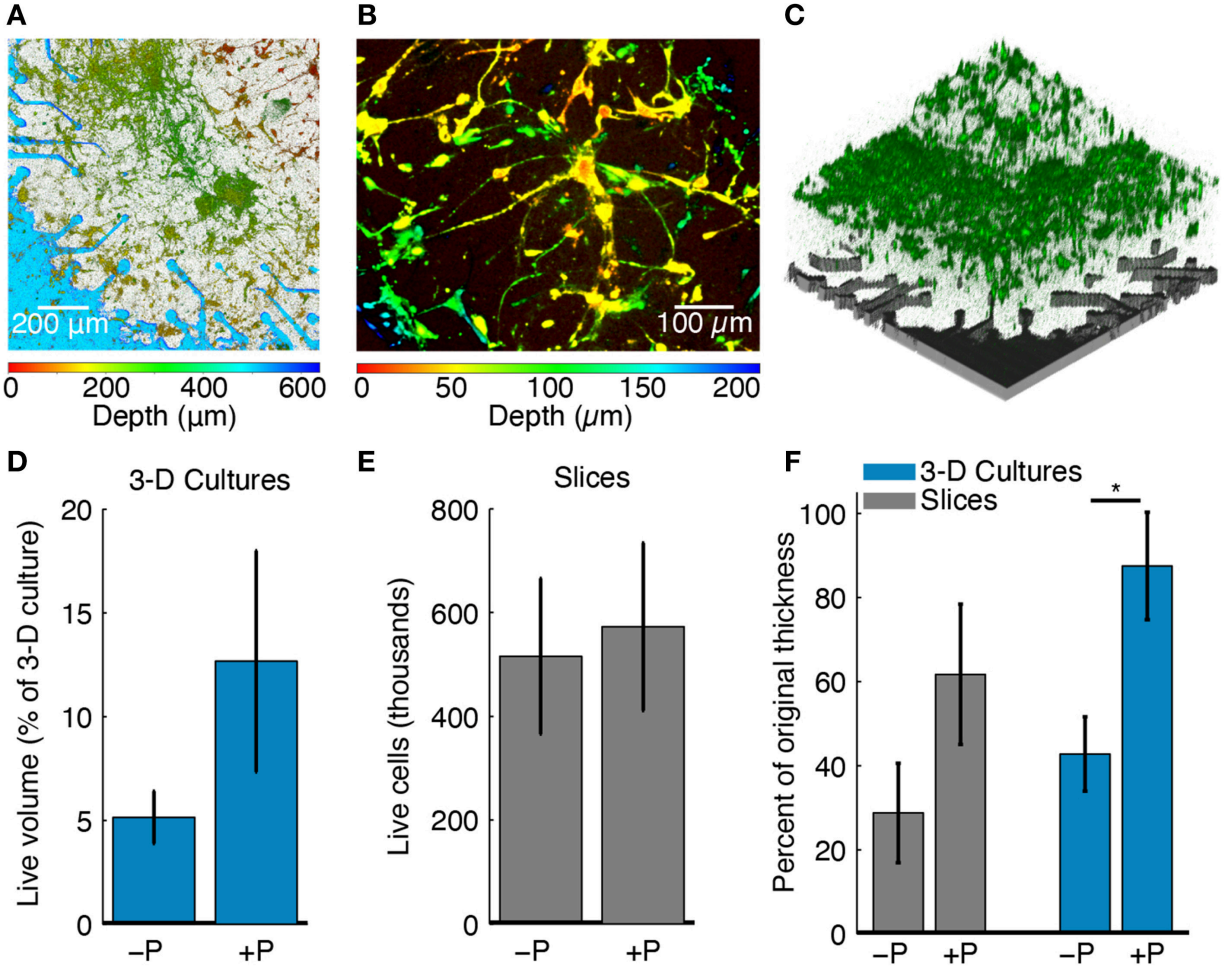
**Effects of perfusion on culture health. (A)** Confocal z-stack through the full 3-D cell culture thickness projected onto a single plane. The color bar below the figure indicates the z-location of live stained cells across the culture thickness. The culture was 500 μm thick at plating and cell depth is shown at 6 DIV. Perfusion maintained good cellular viability and 3-D cell culture thickness. **(B)** A depth-colored projection of a perfused 3-D cell culture showing cells that have grown in three dimensions. **(C)** 3-D rendering of the confocal z-stack in **(A)** taken through the full culture thickness. Live cells stained with an AM dye are shown in green. The location of the pMEA with corresponding perforations and recording sites is shown for reference in gray color. **(D)** Live cell volume fraction for perfused (+P, *N* = 4) and unperfused (−P, *N* = 5) 3-D cell cultures as a percentage of the original plating volume. The blue bars represent the mean value and the error bar range is mean ± SEM. **(E)** Live cell density for perfused (+P, *N* = 4) and unperfused (−P, *N* = 4) brain slice cultures. Slices were pooled from all experiments and normalized by original thickness at plating. The gray bars represent the mean value and the error bar range is mean ± SEM. **(F)** Final culture thicknesses as a percentage of the thickness at plating for perfused (+P, *N* = 4) and unperfused (−P, *N* = 4) slices and 3-D cell cultures (+P, *N* = 4; −P, *N* = 5). Gray and blue bars are represent the mean and the error bar range is mean ± SEM. When perfused, both slices and 3-D cell cultures retained more of their thickness at the end of the experiment (^*^P < 0.05, Wilcoxon rank-sum test). The maximum thickness was taken, which could be greater than the intended thickness at plating due to unevenness at the top surface (see Methods).

Live cells in 3-D cell cultures were stained with an acetoxymethyl (AM) ester dye (either Calcein AM or Fluo-5f AM) and their fluorescence was captured using z-stack confocal microscopy (Figures 4A–C). Each confocal micrograph in the stack corresponded to a specific z-location (depth) within the culture. The entire live cell volume was calculated from respective z-stacks by quantifying fluorescence intensity. First, all images in a z-stack were thresholded to select pixels with intensities above the 80th percentile for each image. This allowed discrimination of fluorescently labeled live cells from the background. Cell survival was then estimated as the volume fraction, i.e., as the volume stained by the AM ester dye divided by the entire culture volume captured in a z-stack.

## Results

Imaging and electrophysiology data were processed and analyzed to assess the effects of perfusion on culture thickness, cell survival, morphology, and functional activity. Figure 4A shows a 3-D cell culture cultured on a perfused pMEA for 6 DIV; this is a planar projection of a z-stack showing neurons and astrocytes distributed in Matrigel ECM. Color-coded cell depth indicating z-distribution of live cell bodies and processes confirmed that the initially 500 μm thick cell culture at plating maintained its initial thickness after 6 days of perfusion on the pMEA. Figure 4B is a thinner and zoomed-in projection of the same culture allowing better visualization of the z-distribution of cells and cellular processes. Figure 4C shows a 3-D rendering of the live cell z-stack in the same culture confirming that indeed live cells (green) were densely packed over the pMEA recording region (gray).

In 3-D cell cultures, the fraction of the culture volume stained with live cell dye (Figure 4E) was used to estimate cell survival. At 6 DIV, perfused 3-D cell cultures had 10.88 ± 4.17% (mean ± SEM) of the volume (the original volume at plating comprising cells and matrix) stained with live cell dye compared to 5.76 ± 1.64% for unperfused 3-D cell cultures. The larger volume fraction of live cells in perfused compared to unperfused 3-D cell cultures suggests that perfusion may have improved cell survival (note that neurons are post-mitotic). The staining of 3-D cell cultures was also more uniform than the staining of slices, owing to significantly lower cell densities in the former than in the latter.

The health of tissue slices was also assessed using cell survival, here defined as the number of live cells in the culture. Cell survival was 11% [(perfused-unperfused)/unperfused] higher in perfused compared to unperfused slices (Figure 4D). To estimate the number of live cells in a culture, cell density in an imaged volume (number of live cells per μL) was multiplied by the final culture volume. Cell counts were then normalized by plating thickness. The imaged volume had a height of ~200 μm over the footprint of the culture chamber. The imaged depth was, on average, 27% of the final thickness of perfused slices and 44% of the final thickness of unperfused slices. In the imaged volume, perfused slices had 8.8 × 10^4^ ± 4.4 × 10^4^ live cells per μL and unperfused slices had 1.7 × 10^5^ ± 7.7 × 10^4^ live cells per μL (mean ± SEM); however, the overall volume of perfused slices was approximately two times greater than that of unperfused slices (see thickness maintenance below and Figure 4F). Assuming that volumetric cell density (calculated number of live cells/μL in the imaged volume) remained approximately uniform throughout the culture thickness, perfused slices had slightly higher cell survival than unperfused slices with an average of 5.73 × 10^5^ cells for perfused slices and 5.16 × 10^5^ cells for unperfused slices (Figure 4D). Considering that we could only effectively stain and image a portion of the slices, estimated cellular densities were consistent with known cellular densities of the rodent brain (Herculano-Houzel et al., 2006), suggesting that a significant fraction of cells survived up to the point of slice imaging. Our estimate of the number of live cells per μl of the imaged volume should also be understood with the following caveats (1) perfused slices were on average twice as thick as unperfused slices, (2) passive diffusion was used for staining and it is likely that thinner (unperfused) slices stained more uniformly than thicker (perfused) slices, and (3) the thinner (unperfused) slices were imaged more completely through the z-thickness without loss of fluorescence signal.

In addition to cellular survival, we examined the maintenance of tissue thickness between perfused cultures and their respective unperfused controls. Thickness maintenance was defined as the ratio between the final culture thickness (at the end of study) and the culture thickness at plating. We found that thickness maintenance was greater in perfused cultures compared to their respective unperfused counterparts, for both brain slices and 3-D cell cultures (Figure 4F). The thickness was, on average, greater in perfused slice cultures by 115% compared to unperfused slices (thickness maintenance of 28.8 ± 11.9% in unperfused vs. 61.8 ± 16.7% in perfused slices; mean ± SEM). Thickness was on average greater by 104% for perfused compared to unperfused 3-D cell cultures (thickness maintenance of 42.8 ± 8.9% in unperfused vs. 87.5 ± 12.8% in perfused 3-D cell cultures; mean ± SEM). The thickness maintenance was significantly greater for perfused 3-D cell cultures (*P* < 0.05, Wilcoxon rank-sum test). Considering both models, perfused cultures had, on average, 104% greater thickness than unperfused controls, while maintaining 75% of the original thickness on average. Indeed, an Analysis of Variance revealed a significant main effect of greater culture thickness with perfusion [*F*_(1, 13)_ = 9.7, P = 0.0083] and no significant effect of culture type (slice or 3-D cell culture). This maintenance of culture thickness by way of interstitial culture perfusion is consistent with previous findings with 700 μm thick brain slice cultures (Rambani et al., 2009).

**Figure 5:**
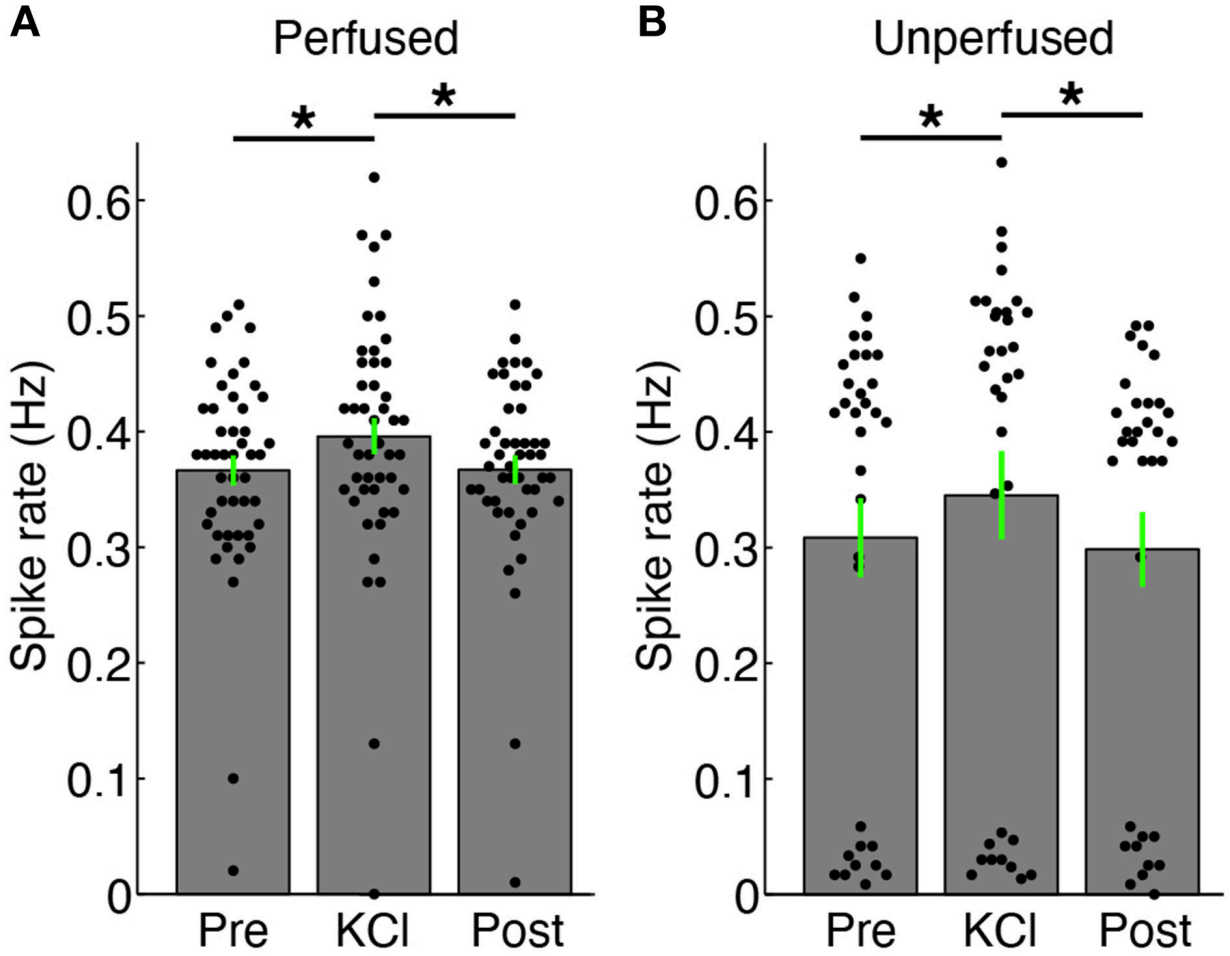
**Changes in firing rate with addition of 20 mM KCl. (A)** Spikes were recorded via substrate electrodes from a perfused 3-D cell culture for a baseline period (“Pre”), with 20 mM KCl medium applied to the surface of the culture, and after a 5X washout of the KCl (“Post”). Each black dot represents the mean spike detection rate on a single electrode. The gray bars are the spike rates averaged over all channels ± SEM. The firing rate of the culture increased upon introduction of KCl solution (^*^P < 0.05, paired *t*-test, *N* = 47 electrodes). **(B)** Spike rates from an unperfused 3-D cell culture recorded under identical conditions as in **(A)**. There were more channels with lower spiking rates in the unperfused culture, and in particular many more channels without active neurons (dots below 0.1 Hz), but the culture also increased its firing compared to baseline (“Pre”) and after washout (“Post”) conditions (^*^P < 0.05, paired *t*-test, *N* = 33 electrodes). There was no significant difference in firing rates between the Pre and Post periods in either case. Fluid was added in the same manner for the KCl and “Post” periods in both perfused and unperfused cultures.

**Figure 6:**
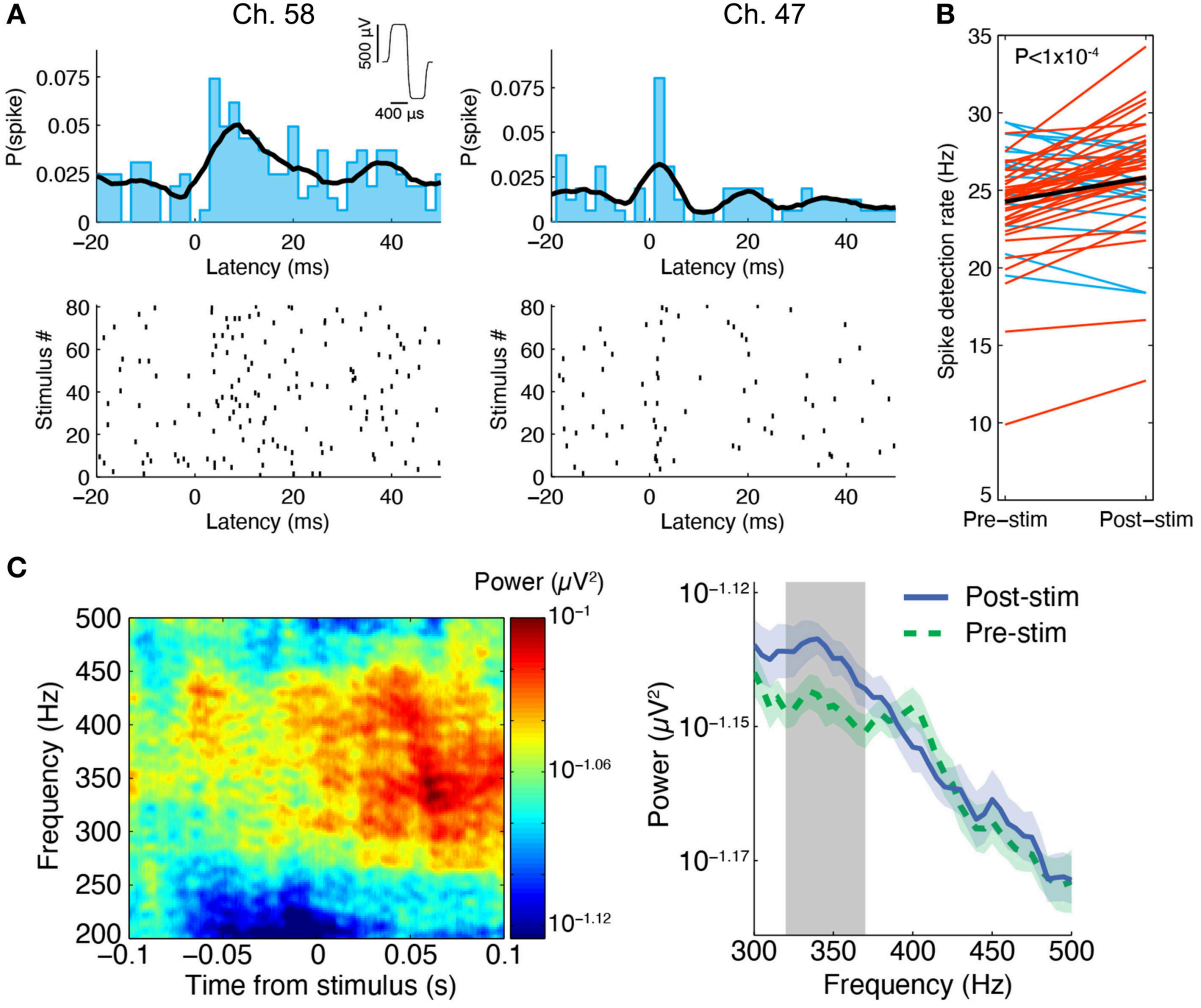
**Stimulus-evoked spiking in perfused 3-D cell cultures at 6 DIV. (A)** Spikes were evoked by electrical stimulation, the results from one experiment in a 3-D cell culture at six DIV are shown here. A stimulus was repeated at 0.83 Hz on a single electrode and it induced neuronal spiking recorded on multiple nearby electrodes, two of which are depicted here. Spikes are shown as black ticks in the raster plot. Each row of the plot represents a different stimulus delivery trial. The spiking probability (the probability of observing a spike at a given time) from 20 ms before to 50 ms after stimulus delivery is shown above the rastergram (2 ms bin width was used, the black curve is the histogram smoothed with a Gaussian, σ = 5 ms). The stimulus waveform was biphasic with 400 μs per phase at ± 500 mV levels (see inset at top). **(B)** Across all active sites in three separate perfused cultures, spike detection rates were higher after stimulation compared to pre-stimulation (paired *t*-test, *P* < 1 × 10^−4^, *N* = 51 sites pooled from three cultures). Red lines represent sites with increased spike detection rates, blue lines represent sites with decreased rates, and the black line represents the mean rate. **(C)** In the same experiment, we found that high-frequency activity between ~250 and 450 Hz was evoked by the stimuli. One example culture is shown here. Left: At an electrode 200 μm from the stimulus site, signal power around 350 Hz increased in the first 100 ms following stimulation (80 ms sliding window, 7 tapers). This can be seen by warm colors in the time-frequency representation of the signal power (μV^2^) on this electrode averaged over all stimulus deliveries. Right: Over all functional recording sites in one culture (*N* = 23 sites), there was a significant increase in signal power near 350 Hz in the first 100 ms after stimulus delivery compared to 100 ms pre stimulus delivery (100 ms sliding window, three tapers; Blue: mean ± SEM post-stimulus power; Green: mean ± SEM pre-stimulus power; Gray: frequency region of significant difference between the two conditions, *P* < 0.05).

We were able to record spontaneous activity from the cultures via the substrate electrodes and to chemically evoke activity using medium with elevated K^+^ concentration. Medium containing 20 mM KCl was applied to the surface of a perfused and unperfused 3-D cell culture at 6 DIV. The elevated KCl significantly increased the firing rate of both cultures (Figure 5). The unperfused 3-D culture had a lower number of active channels than the perfused 3-D culture, which is possibly indicative of fewer live and active cells. Activity could also be elicited from the cultures using electrical stimulation. Figure 6 shows the responses recorded on two electrodes < 750 μm away from an electrode stimulated every 1.2 s with the waveform shown at the top of Figure 6A. Evoked spike events occurred up to 40 ms after stimulation. As shown in Figure 6B, spike detection rates increased in response to electrical stimulation (paired *t*-test, *P* < 1 × 10^−4^, *N* = 51). Stimulation also appeared to evoke high-frequency activity at sites near the stimulation electrode (Figure 6C). Across the entire culture, within 100 ms of stimulation, an increase in signal power around 350 Hz was seen compared to the 100 ms preceding stimulation (Figure 6C; *P* < 0.05, cluster-based permutation test; Maris and Oostenveld, 2007). Interestingly, this is near the peak frequency of the stimulus-induced oscillation described in Hales et al. (2012), suggesting that it may have arisen through a similar mechanism. The oscillation described in Hales et al. (2012) was restricted to the stimulated electrodes, whereas the result described here was observed over a population of recordings sites, suggesting that it may reflect propagating multi-unit activity (MUA). However, there remained a significant increase in high-frequency signal power even after removing the effect of spikes by replacing ±3 ms around spike times (detected at ± 3 standard deviations of the raw signal and resolved at 1 kHz) with the mean value during each snippet. This suggests that the high-frequency signal power may have been independent of action potential waveforms and may have instead arisen from synaptic potentials.

**Figure 7:**
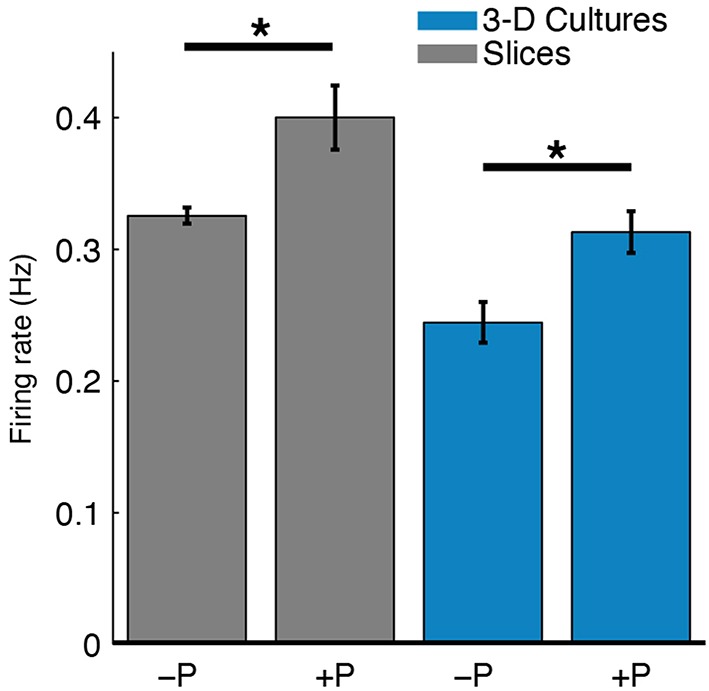
**Perfusion increased spontaneous firing rates of both slices and 3-D cell cultures**. The mean spike detection rate (Hz) per electrode was higher in perfused (+P) cultures compared to unperfused (−P) control cultures (^*^P < 0.05, two-sample *t*-test). A single average firing rate value was taken at each DIV for each culture type. The effect of perfusion was statistically significant as analyzed with 2-way ANOVA (*P* < 0.05; nine slices, six 3-D cultures, see Results for more details). This relatively greater firing rate for perfused cultures may reflect either more live cells near electrodes or greater cellular activity.

Spontaneous spiking activity was recorded daily throughout the experiments. The perfused slices and 3-D cell cultures had higher firing rates than their unperfused counterparts (Figure 7). The firing rates of slices were also higher than in 3-D cultures, consistent with well-developed cells and cellular networks. In 3-D cell cultures, cell densities are significantly lower than in slices and cell-cell signaling and network connectivity was in (relatively) early development. A 2-way Analysis of Variance was performed on the firing rates averaged over all recordings for each culture (unperfused: two slices and two 3-D cultures; perfused: seven slices and four 3-D cultures). Perfusion correlated with a significant increase in firing rates [*F*_(1, 11)_ = 6.6, *P* < 0.05], and slices had overall higher recorded firing rates than 3-D cell cultures [*F*_(1, 11)_ = 8.86, *P* < 0.05]. There was no interaction of perfusion condition and culture type [*F*_(1, 11)_ = 0.61, *P* = 0.45], indicating, as expected, that greater firing rates observed in slices than in 3-D cell cultures are due to better cell network connectivity in the former.

## Discussion

The perfused pMEA interface provided several enabling qualities for electrophysiology research. First, it enabled longer maintenance of functionally more active, thicker sections of explanted tissue and 3-D cell cultures. Next, it enabled maintenance-free, automated culturing for a period of days. Lastly, it enabled distributed electrical recordings, stimulation, and *in situ* culture imaging.

The system was characterized using two tissue models: (a) thick organotypic brain slice cultures, and (b) thick 3-D co-cultures of dissociated cells distributed in an ECM. Although both tissue models are three-dimensional, only organotypic brain slice comprises *ex vivo* circuitry. In both models, development of synaptic connectivity, and to an extent, cell morphology continues to evolve in culture.

Perfusion correlated with an improvement in thickness maintenance, as compared to culture thinning observed in cultures without perfusion. The thinning of unperfused slice cultures is thought to be associated with ischemia and resultant necrosis originating from the culture interior due to limitations in delivery of oxygenated medium intra-culture. This ultimately causes collapse of dying cell layers and reduction in culture thickness (Gähwiler et al., 1997). Convectively enhanced mass transport by way of intra-culture perfusion attempts to mimic *in vivo* circulation (Levick, 1987), providing higher nutrient availability intra-culture, improved removal of catabolites, and gas delivery necessary for maintenance of thick, healthy cultures *in vitro*. It was found that perfusion correlated with greater thickness of slice cultures with respect to unperfused slice controls (62 vs. 29% of the thickness at plating, on average). The same finding extends to 3-D cell culture models of the brain (thickness maintenance of 88 vs. 43% of the plating thickness, on average). While not statistically significant, 3-D cell cultures appeared to maintain thickness better than slices (consistent with lower resistance to mass transport in the former than in the latter). This and previous work suggests that it may be easier to maintain thicker 3-D cell cultures because of their lower cell density, correspondingly lower metabolic need, and lower resistance to intra-culture mass transport compared to slices of explanted tissue of the same thickness.

The significance of perfusion was further assessed by relative cell survival between perfused and unperfused cultures. While 3-D cell cultures exhibited higher live cell densities than their unperfused controls, staining and imaging limitations in thicker (perfused) vs. thinner (unperfused) slices may have underestimated volumetric live cell densities in perfused slices within the imaged volume. However, taking into consideration that perfused slices were on average twice as thick as unperfused slices and had greater cell survival, there appears to be a significant benefit of perfusion. The benefit of perfusion for slice culture is supported by Rambani et al. (2009) who showed that perfused organotypic brain slices were thicker and healthier than their unperfused counterparts. In that study, PI and Hoechst dyes were perfused allowing more uniform slice staining. Next, slices were imaged using two-photon (800 nm) excitation which provided higher signal-to-noise ratio and enabled deeper tissue imaging. Lastly, slices were sectioned in a diametral plane to include culture thickness in z-direction and then imaged. That work revealed that slices plated in 700 μm thickness had over 30% higher viability than both unperfused slice controls and the slices cultured on the membrane inserts (Stoppini et al., 1991; De Simoni and Yu, 2006) at 2 DIV, and ~48 and 51% higher viability than respective unperfused- and membrane insert controls at 5 DIV. Perfused slices were also significantly thicker, maintaining >85% of the initial plating thickness after 5 days of perfusion; a finding that extends to the present study. In the present study, cell survival did not appear to vary significantly in the transverse plane. We did not obtain a vertical distribution of cell survival (across the culture thickness). However, our previous study showed that 5-day perfused brain slices (plated in 700 μm thickness) had cell death only in discrete layers spread across the slice thickness (Rambani et al., 2009).

Since perfusion at prohibitively high flow rates could contribute to culture peeling from the pMEA surface or cell migration from the surface of the array, for all of our studies flow rates were ~3.5 culture volume exchanges per hour for 500 μm thick cultures and ~1.8 culture volume exchanges per hour for 1000 μm thick cultures. These flow rates were below 4.5 volume exchanges per hour at which small fluid channels were previously found to form in brain slices (Rambani et al., 2009).

Notably, both perfused and unperfused cultures in the PDMS-pMEA interface were expected to perform better, in terms of thickness maintenance and cell survival, than a culture in a dish, a culture on an insert (i.e., in an interface-type chamber; Stoppini et al., 1991; De Simoni and Yu, 2006), or a culture on a typical MEA interface without perforations for several reasons. First, the cylindrical culture chamber prevented lateral slice spreading and the corresponding thinning that inevitably occurs in a dish or a membrane insert (Stoppini et al., 1991) due to absence of lateral slice confinement. Second, in contrast to membrane insert cultures (Stoppini et al., 1991; De Simoni and Yu, 2006), in the PDMS-pMEA device slice cultures were entirely submerged in medium. Submersing cultures eliminated surface tension induced disturbances and the corresponding cell damage to slices seated on the insert and the accumulation of bubbles underneath the membrane which often caused discontinuities in basal nutrient delivery. Lastly, the PDMS-pMEA devices doubled the number of culture surfaces exposed to oxygenated medium. Both perfused and unperfused PDMS-pMEA devices provided slices with apical and basal (through the pMEA perforations) access to gas-equilibrated medium whereas a slice-in-a-dish and ordinary MEA setups only provide access, through the culture top. This is significant because ischemia causes rapid decay of slices *ex vivo* and improved access to nutrients and gas mitigates this problem.

The benefit of perfusion to culture health and functional activity was further supported by an increase in detected spontaneous spikes in perfused vs. unperfused cultures (Figure 7). Most recordable cells would have likely been within about 100 microns of the electrode sites (Henze et al., 2000; Claverol-Tinture and Pine, 2002). Hence, the firing rate was a measure of culture health at locations near the electrode array. Here, increased firing rate in perfused cultures may have reflected greater numbers of active cells or increased activity in individual cells. We postulate that perfusion meets metabolic needs of thicker cultures, contributing to greater cell survival and network connectivity. In turn, this may enable electrophysiology studies over a period of days, as shown here, in contrast with acute slice studies lasting only a few hours. Furthermore, while we did not culture longer than 6 days, we expect that longer-term experiments would be possible. For example, 3-D cell cultures created in the same manner have been successfully maintained for over 60 days (Cullen et al., 2011). Slice maintenance, however, is inherently more challenging than maintenance of 3-D cell cultures and future studies with longer-term slice cultures will be important to fully understand the capabilities of the device.

In addition to spike detection, we validated the devices' ability to manipulate functional activity of perfused cultures. As shown in Figure 6C, we detected significant modulation of high-frequency activity induced with electrical stimulation. This suggests that PDMS-pMEA devices may be amenable to studies of population activity such as MUA and field potentials. While field potentials have long been studied in isolated brain slices, they are typically recorded using only a small number of manually inserted electrodes. The PDMS-pMEA device would enable such population activity studies with tissue slices or dissociated cultures in three dimensions with more electrode sites and over longer periods of time than is currently possible. Although difficult to form on a thin film like the pMEA, 3-D electrodes that penetrate the tissue construct would seemingly improve recording and stimulation quality (Rajaraman et al., 2011) and would be especially easy to interface with 3-D dissociated cultures because cells and the ECM are easily cast around three-dimensional templates. Planar electrode arrays, like the pMEA, have been implanted subdurally such that they are close to neurons but typically not close enough to record single units (Leuthardt et al., 2004; Bosman et al., 2012). The planar electrode arrays in these implants are useful for studying population activity and for brain-machine interfaces. Future experiments with the PDMS-pMEA device could capitalize on this analogy to accelerate development of neural implants that would ultimately make use of planar electrode arrays in humans.

For basic science research, the PDMS-pMEA device may augment existing methods for studying population activity. Specifically, the device may be able to resolve: (a) cell activity in a subset of cells in a population, (b) plurality of subsets of cells in a population, and (c) cell activity in mixed cell populations. This can all be done in a 3-D cell culture model *in vitro*, with a greater fidelity to *in vivo* cell morphology and cell network connectivity than in two dimensions. Today, such studies are confined to a small number of electrodes which typically resolve global cell activity or cell activity in a subset of cells at a few discrete locations in a thick culture. This effectively reduces the whole 3-D tissue model to an average cell result, negating the benefit of using physiologically-closer tissue models to study cell function in health and disease. The PDMS-pMEA sandwich interstitial perfusion system enables one to spatially and temporally resolve electrical recordings from subsets of cells in a population, where each subset may surround a specific recording site and where the number of recording sites is measured by multiples of ten, not one. In turn, this allows investigation of specific patterns of cell activity in a subset of cells, or plurality of subsets of the cell population. Collectively, the PDMS-pMEA sandwich interstitial perfusion system allows for a coupled improvement in tissue models and recording fidelity to *in vivo* conditions.

## Author contributions

JV conceived the project. All authors designed the experiments. NK, VV, and JV performed the experiments. NK analyzed the data. NK and JV wrote the manuscript. All authors edited the manuscript.

### Conflict of interest statement

The authors declare that the research was conducted in the absence of any commercial or financial relationships that could be construed as a potential conflict of interest. JV is the president and a shareholder of Lena Biosciences, Inc. The tool was developed by Lena Biosciences, Inc. Experiments were performed at Georgia Tech by NK through a sub-award, and at Lena Biosciences by VV who was employed by Lena Biosciences. VV holds no shares of Lena Biosciences, Inc.
